# Development and evaluation of indirect enzyme-linked immunosorbent assays for the determination of immune response to multiple clostridial antigens in vaccinated captive bred southern white rhinoceros (*Ceratotherium simum simum*)

**DOI:** 10.1186/s13028-020-00555-x

**Published:** 2020-10-07

**Authors:** Angela Buys, Jannie Crafford, Henriette van Heerden

**Affiliations:** 1 Design Biologix Cc, Building 43B, CSIR, Meiring Naude, Brummeria, Pretoria, 0035 South Africa; 2 Department of Veterinary Tropical Diseases, Veterinary Science, University of Pretoria, Onderstepoort, Pretoria, South Africa

**Keywords:** Captivity, *Clostridium*, ELISA, Rhinovax

## Abstract

**Background:**

An overall increase in poaching of white rhinoceros results in captive breeding becoming a significant component of white rhinoceros conservation. However, this type of conservation comes with its own difficulties. When wildlife is captured, transported and/or confined to a boma environment, they are more predisposed to diseases caused by bacterial organisms such as spore forming *Clostridium* spp. A southern white rhinoceros (*Ceratotherium simum simum*) population on a captive bred farm was suspected to be affected by *Clostridium* infections. These endangered animals were apparently exposed to *Clostridium* spp., in the conservation area previously used for cattle farming. The rhinoceros population on the breeding operation property was vaccinated with a multi-component clostridial vaccine registered for use in cattle. Multiple indirect enzyme-linked immunosorbent assays (iELISAs) were developed in order to evaluate the serum antibody titres of these vaccinated animals. In evaluating vaccine efficacy, the gold standard mouse neutralization test (MNT) was not available and therefore iELISAs were developed for the detection of serum antibodies to *C. perfringens* type A (alpha toxin), *C. chauvoei* (whole cell), *C. novyi* (alpha toxin), *C. septicum* (alpha toxin) and *C. sordellii* (lethal toxin) in the white rhinoceros population using international reference sera of equine origin. Antibody titres against each clostridial antigen was evaluated in the vaccinated white rhinoceros population (n = 75). Analytical specificity showed slight cross-reactions for *C. chauvoei* and *C. perfringens* type A with the other antigens. Individual assay cut-off values were calculated with 95% confidence. Coefficient of variance (CV) values for both the international reference sera and in-house control sera across all the antigens were well below 16%, indicating good assay repeatability. This convenient and fast assay is suitable for monitoring humoral immune responses to clostridial antigens in vaccinated white rhinoceroses.

**Results:**

Checkerboard titrations indicated optimal antigen and antibody concentrations to be used for each respective iELISA developed. Each titration set of the respective international reference and in-house control sera showed good repeatability with low standard deviations and coefficient of variance values calculated between repeats for each antigen. Individual assays proved repeatable and showed good analytical sensitivity and specificity.

**Conclusions:**

The developed iELISAs are able to evaluate antibody profiles of phospholipase C, *C. chauvoei* whole cells, TcnA, ATX, TcsL in white rhinoceros serum using international reference sera.

## Background

In this study, a population of captive bred white rhinoceroses experienced episodes of “sudden death” due to a possible infection by one or more species of clostridial organisms. Post-mortem sample evaluation narrowed it down to *C. novyi* and/or *C. sordellii* as the causative agents. The most common pathogenic clostridial species have been well described in livestock in terms of their toxins and pathogenesis [1–4]. In contrast, worldwide, clostridial diseases in rhinoceros are not well documented, with only a few cases described in the literature [5–10]. Consequently, the entire population of white rhinoceroses were vaccinated against multiple clostridial antigens in an effort to reduce mortalities. Currently, no commercial multi-clostridial vaccine is registered for use in wildlife, more specifically, white rhinoceroses. A decision was made to implement the extra-label use of a multi-clostridial vaccine prescribed for use in cattle as an emergency vaccine. However, the efficacy of extra-label use of such a vaccine had to be evaluated. Potency of clostridial vaccines is measured in terms of their ability to induce antibodies against the various toxins or antigens [11] whereas the in vivo mouse neutralization test (MNT) is the statutory method to determine protection by determining the level of antitoxin antibodies against clostridial antigens in the sera of vaccinated rabbits or guinea pigs [12]. This method is however not available in South Africa. Previous studies have reported on the development and evaluation of multiple enzyme-linked immunosorbent assays (ELISA) systems to measure antitoxin in the sera of vaccinated rabbits and compared them to the standard MNT [13–17]. These methods could also be applied to evaluate the exposure of wildlife to multiple infectious agents [18]. Our study aimed to develop and evaluate indirect ELISAs (iELISAs) for the detection of white rhinoceros serum antibodies to antigens of *C. perfringens* type A (alpha toxin), *C. chauvoei* (whole cell), *C. novyi* (alpha toxin), *C. septicum* (alpha toxin) and *C. sordellii* (lethal toxin). The degree of humoral immune response stimulation following vaccination of a multi-clostridial cattle vaccine was assessed.

## Methods

### Study population

A population of white rhinoceroses in a private captive breeding project in South Africa had recently experienced what was diagnosed as a sudden death syndrome with 28 mortalities due to a suspected infection by *Clostridium* spp. The pathology report identified *C. novyi* and/or *C. sordellii* using fluorescent antibody test. Consequently, the population was vaccinated extra-label with a multi-component clostridial vaccine registered for use in cattle. The registered vaccine contained formalinized Al(OH)_3_ adsorbed toxoids or whole cells from *C. chauvoei; C. perfringens* type A, B, C and D; *C. novyi*; *C. septicum*; *C. tetani*; *C. sordellii* and *C. haemolyticum*. All animals received an increased dose of 3 mL via the intramuscular route and were re-vaccinated 4 weeks later. The vaccine is recommended as a 2 mL subcutaneous dose for cattle and sheep. Due to the extreme nature of the mortalities, the on-site veterinarian decided to use an extra-label dose of 3 mL based on the largest volume they were able to inject using remote darting techniques [19]. Blood was collected from 75 animals between 4–12 weeks after the second vaccination and stored as part of the breeding operation serum collection bank. All serum samples evaluated in this study were part of the existing serum collection bank and therefore the study did not require official or institutional ethical approval.

### Checkerboard titrations for iELISAs

Checkerboard titrations were performed in order to simultaneously assess the optimal assay concentrations to be used for both the antibody and the antigen. In order to achieve this, microtitre plates (PolySorp, Nunc, Roskilde, Denmark) were individually coated with each of the five clostridial antigens (Table 1). 100 µL of each antigen at concentrations described in Table 2, was diluted two-fold through row A to row H using 50 mM carbonate buffer, pH 9.6, (Sigma-Aldrich, Saint Louis, MO, USA) and incubated overnight at room temperature. The plates were washed three times with 300 µL per well of TST buffer (0.8 M Tris–HCl, 0.15 M NaCl, 0.05% [v/v] Tween-20; pH 8) to remove unbound antigen (stacked ELISA plate washer, BIO-TEK, Instruments, Winooski, VT, USA). The coated plates were blocked by the addition of 300 µL of blocking buffer (TST buffer with 5% (v/v) fish gelatine (Sigma-Aldrich) at 37 °C for 1 h. After washing three times with TST buffer, 100 µL of the heterologous international reference serum was added in two-fold dilutions through columns 1–12 of the plates, starting with the concentration for each reference serum as indicated in Table 3. Following incubation at 37 °C for 1 h, plates were washed and 100 µL of recombinant protein G conjugated with horseradish peroxidase (Invitrogen), diluted 1:8,000 in blocking buffer, was added to each well. After incubation at 37 °C for 1 h, the plates were washed three times and 100 µL of tetramethylbenzidine (TMB) peroxidase substrate (Invitrogen) was added to each well. The plates were incubated in the dark for 10 min at room temperature (around 25 °C). The reactions were stopped by addition of 100 µL per well of 1 N sulfuric acid and the optical densities (OD) were measured at 450 nm with a BIO-TEK ELISA plate reader. These assays were performed on duplicate plates and repeated on two or three different days. Optimal antigen dilutions were selected in order to coat the plates at the highest antigen dilution that allowed maximum antigen binding capacity. Specific clostridial antigens as described in Table 1 were used to develop individual iELISAs with corresponding international reference sera listed in Table 3. A specific number of units are assigned by the National Institute for Biological Standards and Control (NIBSC), to each of the purchased international reference sera of equine origin as prescribed by the European Pharmacopoeia.

**Table 1:** Summary of the iELISA antigens used for assay development

*Clostridium* species	Source	Antigen
*C. perfringens* type A	Sigma Aldrich	Phospholipase C^b^
*C. chauvoei*	Aphis, USDA*	Flagella antigen
*C. septicum*	Aphis, USDA*	Culture filtrate containing ATX used for MNT by USDA^a^
*C. sordellii*	Aphis, USDA*	Culture filtrate containing TcsL used for MNT by USDA^a^
*C. novyi*	NIBSC (COT)^c^	Culture filtrate containing TcnA used for MNT by USDA^d^

Specific antigens used to coat the plates for the different iELISAs are described as well as their sources

^*^WHO International Standard routinely used for ELISA and or mouse neutralization tests (MNT) donated by the USDA

^a^WHO International ELISA Standard donated by the United States Department of Agriculture (USDA) received as formalinized inactivated antigens

^b^Phospholipase C (P4039, Sigma Aldrich), 125 units per mg lyophilized protein phospholipase C expressed from *C. perfringens* strain 13, plc(988262), chromatographically purified [20]. Phospholipase C may not be the only antigen of importance, but the decision was taken to at least evaluate one target protein per organism based on recommendations in publications. Phospholipase C evaluation was made based on McCourt et al*.* [20]

^c^Internationally prescribed *C. novyi* alpha toxin from the NIBSC as prescribed by the European Pharmacopoeia [21]

^d^National Institute for Biological Standards and Control, NIBSC, received as formalinized inactivated antigens

**Table 2:** Checkerboard titrations

ELISA antigen	Antigen coating dilution	Serum dilution
*C. perfringens* type A	1/3000	1/800
*C. chauvoei*	1/800	1/800
*C. septicum*	1/160	1/100
*C. sordellii*	1/800	1/1600
*C. novyi*	1/3200	1/200

Summary of the checkerboard titrations for the individual iELISAs showing the optimum antigen dilution for coating the plates as well as the serum dilution that falls in the middle of the titration curve

**Table 3:** Assay reference sera

*Clostridium* reference serum	Source	Species of origin	Units for antiserum	Starting concentration of titration curve (Relative units)
*C. perfringens* type A	NIBSC (59/015)	Equine	275	10
*C. chauvoei*	Aphis, USDA	Equine	100	100
*C. septicum*	NIBSC (64/014)	Equine	1100	25
*C. sordellii*	Aphis, USDA	Equine	170	17
*C. novyi*	NIBSC (OE)	Equine	1100	27.5

Specific reference antisera that were used in the individual ELISAs indicating the source, species of origin and the number of units assigned to each serum

*NIBSC* National Institute for Biological Standards and Control, *APHIS* Animal and Plant Health Inspection Service, *USDA* United States Department of Agriculture

### Assay controls

The developed iELISAs were used to screen the 75 rhinoceros sera. Sera with an OD_450_ of ≥ 1.0 ± 0.1 (n = 5) were selected to prepare a pool of positive control sera, and sera with an OD_450_ of ≤ 0.2 ± 0.1 (n = 5) were selected to prepare a pool of negative control sera for each of the individual ELISAs. The dilutions of the in-house positive and negative white rhinoceros serum controls were optimized against each antigen and its respective international reference serum of equine origin following the aforementioned method. During optimization, the IU/mL calculated for the pooled positive sera were diluted by the addition of sterile phosphate buffered saline (pH 7.0) to contain a similar number of units as the reference sera for each respective antigen and used as additional controls on each plate. These reference sera were then titrated in each run of the assays and a 4-parameter logistic curve fitting method was used to assign relative units to rhinoceros sera tested in each assay [22]. Uncoated, blocked plate wells were evaluated in order to establish the background signals for each iELISA.

### Repeatability of the different assays

The repeatability of each of the iELISAs was evaluated using the homologues equine international reference serum as well as a pooled rhinoceros positive control serum. Each serum was tested in duplicate at a high (10 units) medium (1.25 units) and low (0.156 units) concentration on three consecutive days. The intra-assay coefficient of variance (CV) was calculated.

### Testing rhinoceros field sera

For each antigen coated plate, the corresponding control sera were titrated in duplicate in the first four columns in order to get a titration series for each. The starting concentrations for the different control sera are indicated in Table 3. Each test serum was tested at a single dilution in duplicate wells (Table 2). The resulting mean OD_450_ for each test serum was converted to relative units through the implementation of a 4-parameter curve fitting method taking the various dilution factors into account [22].

### Preparation of positive and negative rhinoceros control sera for the individual assays

The white rhinoceros test sera (n = 75) were evaluated in duplicate for each antigen using the pre-optimized conditions (Table 2). An in-house positive serum control was prepared from pooled white rhinoceros serum that showed a mean OD_450_ of 1.0 ± 0.1. Test sera resulting in a mean OD_450_ of 0.2 ± 0.1 were selected to prepare a pooled negative in-house white rhinoceros serum control. The pooled controls were tested relative to the respective international equine reference sera and were adjusted with PBS if the IU/mL value obtained for the pooled serum was more than the highest IU/mL value of the reference serum titration.

## Results

### Checkerboard titrations

The results for the checkerboard titration of the five antigens and homologues equine reference sera for each of the ELISAs are presented in Additional file 1: Figures S1–S5. The optimal antigen dilution to coat the plate, allowing for maximum antigen binding, were selected. A serum dilution that represented a positive sample OD_450_ closest to 1.0 and a negative sample OD_450_ closest to 0.2 was selected for testing the rhinoceros sera. A summary of each antigen and serum dilution chosen based on the results from each checkerboard titration is shown in Table 2. The chosen antigen coating concentrations were relatively low (*C. perfringens* type A: 1/3000; *C. chauvoei*: 1/800; *C. sordellii*: 1/800; *C. novyi*: 1/3200) except for *C. septicum* which was only diluted to 1/160.

### Analytical specificity for the different clostridium iELISAs

Cross reactivity between the different reference antigens and equine reference antisera were evaluated. *Clostridium chauvoei* antigen showed an increase in OD_450_ readings against antisera of *C. sordellii* with 0.206, *C. septicum* with 0.321, *C. novyi* with 0.210 and *C. perfringens* type A with 0.319. *Clostridium perfringens* type A antigen reacted with antisera of *C. sordellii* with 0.176, *C. septicum* with 0.258, *C. novyi* with 0.261 and *C. chauvoei* with 0.319 (Table 4). OD_450_ values for the rest of the cross-reactions were negative with average values correlating to background signals of 0.048–0.066.

**Table 4:** Serum cross-reactions for all *Clostridium* species

	*C. chauvoei antiserum*	*C. sordellii antiserum*	*C. septicum antiserum*	*C. novyi antiserum*	*C. perfringens* type A *antiserum*
*C. chauvoei antigen*	1.357	0.206	0.321	0.210	0.319
*C. sordellii antigen*	0.048	1.058	0.056	0.049	0.176
*C. septicum antigen*	0.086	0.049	1.835	0.055	0.258
*C. novyi antigen*	0.060	0.063	0.084	1.691	0.261
*C. perfringens* type A *antigen*	0.053	0.066	0.062	0.051	1.597

A cross table representing the OD_450_ values of heterologous international reference sera for evaluating cross reactivity between the five clostridial species indicating the analytical specificity of each assays, assisting in the calculation of assay cut-off values

### Analytical sensitivity of the different clostridium ELISAs

The cut-off value for the *C. perfringens* type A ELISA was calculated to be 0.072 OD_450_. This resulted in a minimum level of detection of 0.17 relative units for the assay when considering the heterologous serum mean for *C. perfringens* type A calculated as 0.058 OD_450_ and the addition of 2 SD (SD OD_450_ 0.007) above the mean [23].

The cut-off value for the *C. chauvoei* ELISA was calculated to be 0.393 OD_450_. This resulted in a minimum level of detection of 22.95 relative units for the assay when considering the heterologous serum mean for *C. chauvoei* calculated as 0.264 OD_450_ and the addition of 2 SD (SD OD_450_ 0.065) above the mean.

The cut-off value for the *C. novyi* ELISA was calculated to be 0.310 OD_450_. This resulted in a minimum level of detection of 0.92 relative units for the assay when considering the heterologous serum mean for *C. novyi* calculated as 0.117 OD_450_ and the addition of 2 SD (SD OD_450_ 0.097) above the mean.

The cut-off value for the *C. sordellii* ELISA was calculated to be 0.207 OD_450_. This resulted in a minimum level of detection of 0.51 relative units for the assay when considering the heterologous serum mean for *C. sordellii* calculated as 0.082 OD_450_ and the addition of 2 SD (SD OD_450_ 0.063) above the mean.

The cut-off value for the *C. septicum* ELISA was calculated to be 0.309 OD_450_. This resulted in a minimum level of detection of 0.61 relative units for the assay when considering the heterologous serum mean for *C. septicum* calculated as 0.112 OD_450_ and the addition of 2 SD (SD OD_450_ 0.099) above the mean.

### Repeatability of the different assays

In order to estimate assay variability, the average CV values calculated for each serum titration performed in duplicate wells on the same day and the same assay repeated on different days are presented in Additional file 2: Table S5. Intra-assay repeatability for international equine reference and in-house positive sera of *C. perfringens* type A, *C. novyi* and *C. septicum* were calculated over 3 different days and *C. chauvoei* and *C. sordellii* over 2 different days. CV values for the lowest titres of each antigen were higher than for the higher titres, although still below the acceptable 20% cut-off. Intra-assay repetitions for assay variability revealed standard deviations of between 0.002 and 0.088 across all concentrations of each of the antigens showing good repeatability of the assays for each of the dilutions tested.

### Testing rhinoceros field sera

The white rhinoceros test serum samples were tested at a single dilution as indicated in Table 2 and the relative units are presented in Additional file 3: Figure S6 as a frequency of distribution plot. Of the 75 samples evaluated; 53 samples tested seronegative for *C. chauvoei*; 8 samples tested seronegative for *C. novyi*; 21 samples tested seronegative for *C. sordellii*; 1 sample tested seronegative for *C. septicum*; 1 sample tested seronegative for *C. perfringens type A*. Additional file 3: Figure S6 illustrates the distribution of sample positivity to each antigen tested in order to better portray the sample results.

## Discussion

This study is the first to describe iELISAs that are able to evaluate antibody profiles of phospholipase *C*, *C. chauvoei whole cells*, TcnA, ATX, TcsL in white rhinoceros serum. The ELISAs were developed to determine the clostridial antibody profiles in white rhinoceros sera using standardized international equine reference sera. Each titration set of the respective international equine reference and in-house control sera showed good repeatability with low standard deviations and CV values calculated between repeats for each antigen. Each ELISA was optimized by checkerboard titrations using international equine reference sera against neutralizing epitopes to the target antigens for each organism. The individual iELISAs were specific to homologous reference antisera but some cross-reactivity were detected between *C. chauvoei* whole cells and phospholipase C antigens with the other antisera (Table 4). The target antigens of *C. chauvoei* have been extensively evaluated by several researchers [24–27]. Immunity to *C. chauvoei* is generally associated with antibody to both the bacterium and its toxins, with protection predominantly depending on antibody to cell wall and flagellar antigens [28–30].

Individual iELISAs were applied to 75 sera from a closed, extra-label vaccinated rhinoceros population that had experienced 28 mortalities due to a suspected infection with *Clostridium* spp. Response to vaccination was variable. It was expected that a higher number of animals would show high antibody titres since all the rhinoceros received two commercial cattle vaccinations, four weeks apart. However, a significantly low number of animals showed high antibody titres. It is speculated that incomplete administration of the vaccine, incorrect dosage or route of administration, the iELISA not reacting to the epitopes in the commercial cattle vaccine or a poor immune response was induced in the rhinoceros by this vaccine. Antibodies to phospholipase C, ATX and TcnA may be attributed to the ubiquitous nature of the organism in the gut, immediate environmental exposure or vaccination [31–33]. However, important to note is that eight fully vaccinated white rhinoceroses had died from clostridial infections, three months after receiving two vaccinations with the multi-clostridial cattle vaccine. No serum samples were available for ELISA testing, but liver, heart and soft tissue impression smears tested positive with direct fluorescence antibody tests for *C. novyi* and *C. sordellii*. No serum samples were collected before vaccination with the commercial multi-component clostridial cattle vaccine due to the risks associated with immobilization and handling of the animals. Design Biologix cc was approached after the emergency vaccination of the entire population and no unvaccinated animals were available at that time. Thus, highly reactive positive samples and negative samples in the respective iELISAs were selected and pooled to create negative (low sero-reaction) and positive (high sero-reaction) rhinoceros serums. This is not an ideal study design, but without an alternative option, the positivity and negativity of the rhinoceros sera were correlated with the international reference sera of equine origin. However, this approach of pooling evaluated samples to prepare and validate in-house control samples are accepted and prescribed by the European Pharmacopoeia when international reference sera are used in serological assay validation [12].

Serological tests validated only for livestock species are frequently applied to wildlife samples. The release of animals, either for translocation from one wild population to another, the introduction of captive-bred animals into a natural wild population, or the return of rehabilitated animals into the wild after varying periods of time in captivity, have become conventional and may contribute to diseases in wildlife. Thus, the risks of disease transfer must be assessed as these animals may harbour pathogenic viruses, bacteria, protozoa, helminths and arthropods. Some organisms may become pathogenic when the host undergoes stressful situations, affecting not only the released animal but equally important, other animals, including humans, in the environment [34, 35]. There is a general deficiency of information relating to the incidence of disease and/or the pathogenicity of specific agents for most species of African wildlife. Health screening of animals, pre-translocation as well as the indigenous wildlife in the reception area should be well defined. However, interpretation of unvalidated serological test data of wildlife with serological tests developed for domestic livestock species due to a lack of species-specific reagents, standardization and availability of positive and negative sera should be done with caution. From a wildlife management perspective, this can lead to false positive and negative results with serious implications for epidemiological surveillance. The development of assays specific for wildlife will provide reliable information that can be interpreted with greater confidence and insight.

Whilst toxin neutralisation tests are known to be sensitive and specific, they have the following disadvantages: time consuming, imprecise results, expensive, require the use of large numbers of laboratory animals and animal use and care issues (impact on animal welfare). These assays have severe adverse effects on the laboratory animals which vary from paralysis to death. Therefore, alternative assays are being investigated for the evaluation of vaccine potency against these organisms and their toxins [16, 17, 36]. The unavailability of a validated gold standard MNT in South Africa accentuated the need for the development of the current iELISAs for vaccine efficacy evaluation. However, a correlation study of the iELISAs and the MNT will have to be considered in future, as antibody response to vaccines may differ in different species [37]. The data generated by the ELISAs indicates sero-conversion and not protection upon vaccination. The prescribed minimum vaccine requirements (IU/mL, Ph. Eur.) use protective cut-off values for the MNT with vaccinated rabbit or guinea pig sera in mice that might differ from white rhinoceros sera in mice. Due to current restrictions in South Africa, the test will have to be outsourced to facilities capable of performing the validated MNT outside South Africa. These studies may prove to be difficult due to the limited availability of serum samples with known vaccination histories from wildlife species as well as the practicality of handling larger numbers of wildlife.

For the iELISA to be considered as a viable means for serological evaluation, further validation of the assay is required in order to ensure fitness for purpose. Although the current developed assays were capable of distinguishing between serums with varying degrees of positivity, the developed assays are only capable of detecting the presence of serum antibodies directed to the various epitopes of the crude antigens coating the plates. This is only an indication of exposure to the antigens, whether natural or prophylactic, and not a measure of protection provided by the vaccine. Correlation studies are therefore required to compare the protective antibody titres of the European Pharmacopoeia prescribed MNT to the different iELISAs titres, using pre- and post-vaccination white rhinoceros sera. In addition, the evaluation of larger numbers of samples may be beneficial to the implementation of the developed iELISAs for vaccine potency evaluations, however, the availability of white rhinoceros serum samples with known vaccination histories are extremely limited. This can however be overcome by vaccinating and testing wild calves older than 6 months born to unvaccinated mothers.

As an alternative to optimising the current iELISA, consideration may also be given to the use of a competitive ELISA in future studies. Several reports have demonstrated a high correlation (at least 0.93) in antitoxin levels determined by competitive ELISA, based on the use of a monoclonal antibody and the MNT [13, 16, 38, 39]. However, monoclonal antibodies are not always readily available to most clostridial antigens. This could be resolved if the EDQM make monoclonal antibodies available to the industry for in vitro assay validation for all the veterinary important clostridia and allow for better correlation to the current MNT. Alternatively, the production of recombinant target antigens for each organism might increase the sensitivity and specificity of the developed iELISA assays [40, 41]. Collaborative studies should be done with international laboratories capable of performing a validated mouse neutralization test together with the use of possible recombinant antigens for iELISA development in order to overcome the current challenges experienced by clostridial research in South Africa.

## Conclusions

The developed assays in this study proved to be a useful tool in the evaluation of seroconversion to the five clostridia evaluated after vaccination. Although antibody prevalence is not an indication of protection against disease it can be a valuable tool for the monitoring of vaccination programs in wildlife species in extensive captive breeding programs. It can also aid in assessing the risk of translocation of possibly unexposed wildlife into infected environments. However, whilst toxin neutralisation tests are known to be sensitive and specific, they have many disadvantages: time consuming, imprecise results since protection is expressed as a range, expensive, require the use of large numbers of laboratory animals and animal use and care issues. Mouse assays have severe adverse effects on the host which vary from paralysis to death. Therefore, data generated by the suggested alternative assays from this study justifies further investigation for their use in evaluation of vaccine potency and diagnostics against these clostridial organisms and their toxins.

## Supplementary information

**Additional file 1.** Analytical sensitivity of the assays for this study is included as additional data.

**Additional file 2.** Repeatability of the assays for this study is included as additional data.

**Additional file 3.** Frequency distribution of the data for this study is included as additional data.

## Data Availability

The datasets used and/or analysed during the current study are available from the corresponding author on reasonable request.
