# Supplementary material for: Development and evaluation of indirect enzyme-linked immunosorbent assays for the determination of immune response to multiple clostridial antigens in vaccinated captive bred southern white rhinoceros (Ceratotherium simum simum)
Source: Acta Vet Scand. 2020 Oct 7;62:57. doi: 10.1186/s13028-020-00555-x (PMC7541252; doi:10.1186/s13028-020-00555-x)
Supplement: Supplementary file 1 — Additional file 1. Analytical sensitivity of the assays for this study is included as additional data. [file 13028_2020_555_MOESM1_ESM.docx]

**Additional File 1** Analytical sensitivity of the assays for this study is included as additional data.

**Figure S1.** The cut-off value for *Clostridium perfringens* type A was calculated with 95% confidence in order to demonstrate the analytical sensitivity of each assay. A 2-fold dilution range was prepared for each serum. The cut-off value for *C. perfringens* type A was determined to be 0.058 OD_450_ or 0.17 relative units as indicated by the arrow on the graph.

**Figure S2.** The cut-off value for *Clostridium chauvoei* was calculated with 95% confidence in order to demonstrate the analytical sensitivity of each assay. A 2-fold dilution range was prepared for each serum. The cut-off value for *C. chauvoei* was determined to be 0.393 OD_450_ or 22.95 relative units as indicated by the arrow on the graph.

**Figure S3.** The cut-off value for *Clostridium novyi* was calculated with 95% confidence in order to demonstrate the analytical sensitivity of each assay. A 2-fold dilution range was prepared for each serum. The cut-off value for *C. novyi* was determined to be 0.310 OD_450_ or 0.92 relative units as indicated by the arrow on the graph.

**Figure S4.** The cut-off value for *Clostridium sordellii* was calculated with 95% confidence in order to demonstrate the analytical sensitivity of each assay. A 2-fold dilution range was prepared for each serum. The cut-off value for *C. sordellii* was determined to be 0.207 OD_450_ or 0.51 relative units as indicated by the arrow on the graph.

**Figure S5.** The cut-off value for *Clostridium septicum* was calculated with 95% confidence in order to demonstrate the analytical sensitivity of each assay. A 2-fold dilution range was prepared for each serum. The following cut-of value for *C. septicum* was determined to be 0.309 OD_450_ or 0.61 relative units as indicated by the arrow on the graph.
