# Supplementary material for: Development and evaluation of indirect enzyme-linked immunosorbent assays for the determination of immune response to multiple clostridial antigens in vaccinated captive bred southern white rhinoceros (Ceratotherium simum simum)
Source: Acta Vet Scand. 2020 Oct 7;62:57. doi: 10.1186/s13028-020-00555-x (PMC7541252; doi:10.1186/s13028-020-00555-x)
Supplement: Supplementary file 2 — Additional file 2. Repeatability of the assays for this study is included as additional data. [file 13028_2020_555_MOESM2_ESM.docx]

**Additional File 2** Repeatability of the assays for this study is included as additional data.

**Table S5.** The repeatability of each of the iELISAs was evaluated using the heterologous international equine reference serum as well as a pooled rhinoceros positive control serum. Each serum was tested in duplicate at a high (10 units) medium (1.25 units) and low (0.156 units) concentration on 3 consecutive days.
