# Supplementary material for: Development and evaluation of indirect enzyme-linked immunosorbent assays for the determination of immune response to multiple clostridial antigens in vaccinated captive bred southern white rhinoceros (Ceratotherium simum simum)
Source: Acta Vet Scand. 2020 Oct 7;62:57. doi: 10.1186/s13028-020-00555-x (PMC7541252; doi:10.1186/s13028-020-00555-x)
Supplement: Supplementary file 3 — Additional file 3. Frequency distribution of the data for this study is included as additional data. [file 13028_2020_555_MOESM3_ESM.docx]

**Additional File 3** Frequency distribution of the data for this study is included as additional data.

**Figure S6.** Frequency of distribution plots representing white rhinoceros serum samples tested with the individual iELISAs 3 months after they were vaccinated with two doses (4 weeks apart) of a commercial multi-component clostridial vaccine for cattle. The cut-off values are indicated with an arrow on the x-axis.
